# An examination of user costs in relation to smokers using a cessation service based in the UK

**DOI:** 10.1186/s12913-018-2985-1

**Published:** 2018-03-15

**Authors:** Neil Walker, Yaling Yang, Vasiliki Kiparoglou, Subhash Pokhrel, Hayley Robinson, Hugo van Woerden

**Affiliations:** 1 NIHR Oxford Biomedical Research Centre, Churchill Hospital, Old Road, Headington, Oxford, OX3 7LE UK; 2 Nuffield Department of Primary Care Health Science, University of Oxford, Radcliffe Primary Care Building, Radcliffe Observatory Quarter, Woodstock Road, Oxford, OX2 6GG UK; 3 Health Economics Research Group, Institute of Environment, Health and Societies, Brunel University, Uxbridge, UB8 3PH UK; 4 Quit 51 Stop Smoking Service, Burton-on-Trent, DE14 2AZ UK; 5 Institute of Primary Care & Public Health, Cardiff University, Cardiff, UK; 6 Centre for Health Science, University of the Highlands and Islands, Inverness, IV2 3JH UK

**Keywords:** Smoking cessation, Cost per quit, NRT, Varenicline

## Abstract

**Background:**

Smoking cessation services provide support to smokers who desire to quit. Published studies to date have looked at the cost and benefit of service provision but typically focus on clinical trial data. Using routinely collected observational data, this study examined the costs involved in providing a service in terms of average health care expenditure per successful quit attempt in addition to population – level cost-effectiveness measures.

**Methods:**

Data were analysed from Quit-51 smoking cessation service across five English regions between March 2013 and March 2016 (*n* = 9116). For each user, costs were estimated in relation to: (i) time spent with advisers; (ii) prescription of pharmacotherapy. The total costs compared against self-reported quit at 12 weeks, which represents the time period for which the service is offered. Cost per quit (CPQ), with 95% confidence interval (CI), was calculated by relating total expenditure to the number of quitters, firstly for the whole dataset and then by subgroups of key categorical variables, namely; gender, age group, the Fagerstrom test for nicotine dependence (FTND) and Index of Multiple Deprivation (IMD). Confidence intervals (CIs) for the mean estimates were derived using a non-parametric bootstrap procedure. Parameters derived from the calculation in relation to treatment were used to estimate potential long-term population outcomes under a scenario where the Quit 51 prescription was rolled out nationally.

**Results:**

The overall mean CPQ for this sample as estimated at 12 weeks was £403.51 (95% CI = £393.36 to £413.76). The estimated CPQs at this time point were comparable for those aged 12–19 (£423.56, 95% CI = £369.45 to £492.60) and those aged 20–29 (£430.76, 95% CI = £395.95 to £470.56). Differences were also seen in relation to other subgroups considered. The treatment parameters translated to a projected increase of 1.5 quality-adjusted life years (QALYs) per 1000 smokers in the short-term and 23.4 QALYS per 1000 smokers based on a lifetime horizon.

**Conclusions:**

These figures throw light on service expenditure for each successful quit over the timeframe for which the service is offered in addition to highlighting variability in these costs across different subgroups of the user population.

## Background

Smoking is a major cause of preventable death and poor health in countries across the world [1]. In particular, smoking is linked to lung and other cancers [2] as well as heart disease, stroke and a range of pulmonary conditions. Bans on smoking in public places, coupled with increasing awareness of the adverse effects on health have helped to reduce the number of smokers in many countries [3]. As well as being beneficial to public health, reducing the prevalence of smoking relieves pressure on health services as a result of the consequent drop in smoking related admissions and achieving further reduction remains a priority for healthcare providers around the world [4].

In the United Kingdom, support is offered to those wishing to quit through the provision of smoking cessation services which operate according to guidelines laid out by the National Centre for Smoking Cessation Treatment (NCSCT) based on the latest available evidence on effective cessation strategies [5, 6]. Similar services exist in other countries [7]. Smoking services provide access to advice over a number of sessions with a qualified adviser and tailored pharmacotherapy in the form of Nicotine Replacement Therapy, or NRT [8], which is offered for this purpose. Varenicline (proprietary name in UK - Champix) and bupropion (proprietary name in UK - Zyban) are also available, although the last of these is rarely used in light of concerns over side-effects [9].

Of these three treatments, varenicline consistently demonstrates superiority with respect to quit rate based on results from clinical trials [10, 11], although alternative treatments not currently available in the UK, e.g. cytisine, may be more effective still [12]. It is less clear whether bupropion is superior to NRT *or* vice versa, although it is well established that both perform well in relation to placebo [13]. Whilst these findings appear to hold over a number of studies, the projected quit rates and cost effectiveness have not been fully characterised and the decision to prescribe a particular treatment over alternatives may be based on the preference of clinical staff rather than an analysis of client characteristics.

In a climate where healthcare expenditure is increasing in line with new technologies and greater demand, such decisions should be informed by consideration of costs and benefits [14]. Economic models of these smoking cessation interventions from a number of studies indicate that varenicline is the most cost-effective according to various measures such as Quality Adjusted Life Years (QALYs), cost savings and reduction in incidences of smoking-related mortality [15–17]. Results with respect to pharmacotherapy are highly variable when compared across different studies and countries [18]. The costs and benefits used in models of the cost effectiveness of smoking cessation interventions are typically derived from clinical randomised controlled trials (RCT). It is well recognised that the cost-effectiveness of a given intervention can be different in a real-world as opposed to a research context [19–21].

Our primary aim was to calculate the cost per quit (CPQ) in relation to the above treatments using data collected by a smoking cessation service, which affords an insight into the costs in routine clinical practice. We also carried out CPQ calculations across key subgroups in the population of smoking cessation service users. As a secondary aim, figures arising from these calculations, in relation to the three pharmaceutical treatments, were utilised in order to calculate what the long-term population-level benefits may be if the Quit 51 programme were rolled out nationally using a freely available tool designed for the calculation of long-term population health parameters, the EQUIPT calculator [22].

## Methods

### Smoking cessation services

Quit 51 offer a smoking cessation service in accordance with National Institute for Health and Care Excellence (NICE) guidelines [6]. The remit of the service is to provide a maximum of 12 sessions of support with an accredited adviser and provision of tailored pharmacotherapy to smokers attempting to quit. A session is typically of 15 min duration although the introductory session will generally take longer in order to cover triaging and discussions around individual background and requirements. Assuming a patient continues with the service for the full duration, they should receive a minimum of 90 min’ contact time with an adviser covering a period up to 12 weeks after quitting.

### Data

Data were provided by Quit 51 on patients across six Clinical Commissioning Groups (CCGs) in England for whom the requisite information on treatment and contact time was available. The period covered varied between the different CCGs, but all records fell within the dates 15 March 2013 to 16 March 2016 inclusive (using quit date as the criterion). Best practice was observed in terms of data handling and the project conformed with the principles laid out in the World Medical Association Declaration of Helsinki [23].

At the outset, there were 15,682 records, but a number of restrictions were applied before calculations were made. Observations were removed where: (i) quit date was before subsequent to 9 December 2015, *N* = 1950 (ii) smoker recorded as pregnant, *N* = 802 (iii) either no or multiple pharmacotherapy was recorded, *N* = 4254. The first of steps was taken to ensure as far as possible that all records were complete at the date of data compilation (March 13 2016). Pregnant users of Quit 51 were not considered here as this subgroup is offered a specialist service which differs in key respects from the standard service. Instances where no or multiple treatment were omitted due to a possibility of misrecording. The above restrictions gave rise to a dataset of 9116 observations derived from 7783 individuals. In order to gain a global perspective on CPQ over this period, repeat observations on individual clients were retained in the calculation. Of the original six clinical regions considered (East Sussex, Sandwell, Surrey, Telford and Wrekin, Walsall, Worcester) only five were used in this calculation as all the Surrey records fell after the exclusion date of 9 December 2015.

### Individual costs

A cost for each individual using the service was estimated based on: (i) pharmacotherapy prescribed; (ii) time spent with an adviser.

With respect to pharmacotherapy, the costs, including prescription and value added tax (VAT) for each treatment were as follows: NRT (combination) - £19.95 per week; Varenicline - £76.80 per month/£38.40 per 2 weeks; and Bupropion - £69.45 per month/£34.73 per 2 weeks. For clients using NRT, treatment was made available for use to coincide with the agreed quit date. Varenicline, on the other hand, was prescribed between 8 and 14 days prior to the quit date in order to allow for the medical properties of this treatment to take effect. Sometimes an anomaly was observed whereby prescription of these treatments occurred after the 12-week treatment period. Since our inference was based on cessation at 12 weeks, prescription costs were only considered within this timeframe. Table 1 describes how doses were prescribed over time according to treatment pathway. The derivation of medication costs was assessed according to the number of doses prescribed and the individual dose quantities (costs for dose quantities provided above). If, as in some instances, the number of doses indicated in the dataset was above the maximum specified in Table 1, for the purposes of the calculation this was fixed at this maximum, so as not to include costs beyond the standard treatment timeframe.

**Table 1:** Chronological pattern of medication dose (in weeks) for users of Quit 51 smoking cessation service according to pharmacotherapy prescribed and the prescription route (through pharmacy or other)

Treatment (prescription pathway)	Chronological pattern of dosing covering 12 weeks of service use (dose quantity in weeks)	Max. no. of doses
NRT (all)	1 + 1 + 1 + 1 + 1 + 1 + 1 + 1 + 1 + 1 + 1 + 1	12
Varenicline (pharmacy)	2 + 2 + 2 + 2 + 2 + 2	6
Varenicline (any route other than pharmacy)	2 + 2 + 4 + 4	4
Bupropion (all)	2 + 2 + 4	3

The time spent with an adviser was recorded for each session attended. The figures around adviser time for each session were based on average values, although it is conceivable that a degree of variability exists on a case-by-case basis, for example, in relation to distance travelled by an adviser. The total time spent with an adviser was taken from a variable called “Total Contact Time” in the master database, covering the full 12 weeks of potential engagement with the service. The estimated time was multiplied by an hourly charge of £26.32, based on the following breakdown of costs: (i) Adviser - £23.65 per hr.; (ii) Room - £0.20 per hr.; (iii) Equipment - £0.49 per hr.; (iv) Travel - £0.74 per hr.; and (v) Advertising - £1.24 per hr. This itemisation of costs in relation to both pharmacotherapy and counselling was given by the service provider, based on the financial year 2015–2016. Central overhead costs for the service were not included.

### Cost-per-quit calculation and confidence intervals

Using the above method, we derived individual costs up to the full potential 12 weeks’ of service use. As an outcome we used 12-week self-reported quit. This was recorded as a binary response (0 or 1) where 0 indicated ‘failed quit attempt’ and 1 indicated ‘successful quit attempt’. The average CPQ for this dataset was calculated at this time point as follows:

$$ CPQ=\frac{T}{n_{quit}} $$

where *CPQ* = the average CPQ in the sample, *T* = the total costs summed across all patients and *n*_*quit*_ = the total number of successful quits (i.e. where binary outcome of quit success = 1). The average CPQ was then also calculated for all categories of the following factors: (i) Age, (ii) Gender, (iii) Treatment, (iv) Fagerstrom test for nicotine dependence (FTND) index [24] and the Index of Multiple Deprivation (IMD) [25]. Age was split into arbitrary categories (< 19, 20–29, 30–49, 50–69 and 70+ years) for this purpose.

The FTND index is a score (1–10) reflecting an individual’s degree of dependence on cigarettes, based on a number of questions. The lower the score, the lower the dependence inferred. To simplify presentation, this was re-categorised into four groups (0–3, 4–5, 6–7 and 8–10) prior to analysis such that each category consisted of a suitable number of records for statistical purposes. The IMD is an area deprivation score mapped to postcode and scored on a scale of 1–10 inclusive, with lower scores indicating greater deprivation. In order to facilitate interpretation when referring to a deprivation effect, these scores were inverted so that 1 = least deprived/most affluent area with increasing score indicating a greater degree of deprivation (maximum = 10). As with FTND, this measure was grouped into suitably large categories prior to the calculation (deprivation = 1–3, 4–6, 7–8 and 9–10 respectively).

Derivation of confidence intervals was complicated by the fact that the distribution of costs was non-standard, in addition to the fact that there were two populations to take into account - successful quits and failed quits. Thus there were different sources of uncertainty to contend with: (i) the costs within both the failed and successful quit subgroups; and (ii) the proportion of successful/failed quits.

To avoid assumptions about the underlying distributions a bootstrap procedure was used to simultaneously deal with both sources of uncertainty. For a given simulation, the original population was sampled with replacement N times where N = the number of observations in the population. If a given record was sampled, both the cost and quit status were retained. From this bootstrap sample, a CPQ was calculated as above (dividing the total cost by the number of successful quits). This procedure was repeated 1000 times giving a bootstrap sample from which a 95% confidence interval was derived with reference to the percentiles at 2.5% and 97.5%. This procedure was carried out within each subgroup with the value N being determined by the relevant subgroup sample size.

All the CPQ calculations were carried out in GenStat [26] 18th edition.

In order to extrapolate the short-, medium- and long-term healthcare costs and outcomes (QALYs) of Quit 51, a recently developed return on investment (ROI) model, known as EQUIPTMOD [22], was applied using the treatment parameters used here. The EQUIPTMOD is a European initiative to quantify the utility on investment in protection from tobacco [27] and allows users to simulate the costs and QALYs of smoking cessation interventions under various user-defined scenarios. Details regarding the EQUIPT model and underlying assumptions are described elsewhere [22] and the software is available at the following website (http://equipt.eu).

Extrapolating from the five regions represented in our calculation, a scenario was assumed in which Quit 51 was rolled out to the whole of England. This was then compared against a baseline scenario in which no smoking cessation support was available. The baseline thus represented theoretical gross cost of tobacco smoking in the absence of interventions. The difference in costs (both treatment costs due to smoking attributable diseases and costs of implementing Quit 51 at the national level) and QALYs could then be regarded as the benefit derived from the Quit 51 service. In order to achieve this, the EQUIPTMOD was first run according to a “baseline” setting of no treatment. Then, we added parameters based on the ‘Quit 51 approach’ to the model via three specific interventions that characterise the Quit 51 service (and Stop Smoking Services more generally), i.e. NRT + support; varenicline + support; and bupropion + support, using the ‘customise interventions’ functionality of EQUIPTMOD. Three parameters were entered for each treatment arm to model the Quit 51 approach in EQUIPTMOD, namely the uptake rate amongst quitters, the cost per user of the service (regardless of whether the individual has quit or not) and projected relative rate of abstinence (relative to no treatment baseline of 4%) at 12 months. The values entered for uptake and cost per head reflected those in our sample, whereas the relapse rates were based on 12-month estimates as used in EQUIPTMOD for users of NRT, varenicline and bupropion (albeit without behavioural support as is provided in the Quit 51 prescription).

## Results

Summary statistics in Table 2 highlight trends in quit rates between different subgroups before we consider results from statistical analysis. Frequency of prescriptions shows that NRT was prescribed for more than 80% of the clients in this sample and also highlights the rarity with which bupropion was prescribed (0.4% of clients).

**Table 2:** Summary statistics for key variables in the sample based on quit rate at 12 weeks (*N* = 9116)

		*N* (%)	N quit at 12 weeks (%)
Age (mean = 44.8 yrs)	12–19 yrs	509 (5.6%)	116 (22.8%)
	20–29 yrs	1189 (13.1%)	296 (24.9%)
	30–49 yrs	3911 (43.0%)	1262 (32.3%)
	50–69 yrs	2955 (32.5%)	1068 (36.1%)
	70+ yrs	538 (5.9%)	192 (35.7%)
Gender	Male	4249 (46.6%)	1425 (33.5%)
	Female	4867 (53.4%)	1510 (31.0%)
Treatment	NRT	7373 (80.1%)	2117 (28.7%)
	varenicline	1708 (18.7%)	799 (46.8%)
	bupropion	35 (0.4%)	19 (54.3%)
FTND (Mean = 4.89)	0–3	1534 (26.2%)	622 (40.5%)
	4–5	1884 (32.2%)	727 (38.6%)
	6–7	1676 (28.6%)	641 (38.2%)
	8–10	766 (13.1%)	236 (30.8%)
Deprivation (Mean = 7.22)	1–3	886 (10.8%)	319 (36.0%)
	4–6	1838 (22.4%)	635 (34.5%)
	7–8	2157 (26.3%)	698 (32.4%)
	9–10	3321 (40.5%)	1180 (35.5%)

The overall average CPQ was £403.59 (95% CI = £393.36 to £413.76) at 12 weeks (Table 3). Differences were observed between age groups such that CPQ tended to decrease with increasing age, although for the two youngest age groups (12–19 and 20–29 years) the average costs were effectively indistinguishable when the CIs are taken into account (mean CPQs; £423.56 and £430.76 respectively) as was the case also for the two oldest age groups (mean CPQ = £393.22 and £390.88 for the 50–69 and 70+ year age groups respectively). Regarding the pharmacotherapy treatment groups, varenicline use was associated with a slightly higher CPQ than NRT use (mean CPQ = £401.60 and £413.06 for varenicline and NRT respectively), but overlapping confidence intervals for these estimates implied insufficient evidence to conclude that this represented a significant difference. The cost per quitter for smokers prescribed bupropion was markedly lower than for the other two treatments (mean CPQ and 95% CI; £226.56, £177.21 to £314.21).

**Table 3:** Average cost per quit (with approximate 95% CI) calculated at the 12 week time point, with supporting information

Variable	Levels	12 weeks					
		N	Total cost (£)	Cost per head (£)	N quits	Quit rate (%)	Mean CPQ (95% CI) (£)
Age	12–19	509	49,133	96.53	116	22.79	423.56 (369.45, 492.60)
	20–29	1189	127,504	107.24	296	24.89	430.76 (395.95, 470.56)
	30–49	3911	512,284	130.99	1262	32.27	405.93 (391.53, 423.23)
	50–69	2955	419,963	142.12	1068	36.14	393.22 (378.45, 409.60)
	70+	538	75,050	139.50	192	35.69	390.88 (357.16, 427.00)
Gender	Male	4249	561,404	132.13	1425	33.54	393.97 (380.36, 407.97)
	Female	4867	623,127	128.03	1510	31.03	412.67 (398.41, 427.01)
Treatment	NRT	7373	850,191	115.31	2117	28.71	401.60 (389.91, 414.63)
	varenicline	1708	330,035	193.23	799	46.78	413.06 (395.16, 433.55)
	bupropion	35	4305	122.99	19	54.29	226.56 (177.12, 314.21)
FTND	0–3	1534	236,567	153.62	622	40.55	378.87 (359.35, 399.99)
	4–5	1884	299,362	158.90	727	38.59	411.78 (393.08, 433.31)
	6–7	1676	270,188	161.21	641	38.25	421.51 (401.06, 444.88)
	8–10	766	120,191	156.91	236	30.81	509.28 (464.80, 562.87)
Deprivation	1–3	886	136,039	153.54	319	36.00	426.45 (396.92, 460.57)
	4–6	1838	266,099	144.78	635	34.55	419.05 (398.11, 443.28)
	7–8	2157	300,324	139.23	698	32.36	430.26 (408.13, 451.16)
	9–10	3321	451,353	135.91	1180	35.53	382.50 (369.26, 397.69)

Our calculations showed an elevated average cost for females relative to males (mean CPQ = £412.67 and £393.97 respectively) with the confidence intervals indicating that this difference was statistically significant.

The costs steadily increased in line with increased tobacco dependence (mean CPQ = £378.87, £411.78, £421.51 and £509.88 for binned categories of FTND; 1–3, 4–5, 6–7 and 8–10 respectively). The trend with respect to IMD scores was not obviously linear. The CPQ for the first 3 groups, in ascending order of deprivation, were broadly similar (£426.45, £419.05 and £509.88 for deprivation score groups of 1–3, 4–6 and 7–8 respectively). In contrast, the average CPQ for the group of highest deprivation (inverted IMD = 9–10) was £382.50, significantly lower than all others.

Table 4 reports the results from the analysis based on the EQUIPTMOD. Parameters entered to simulate a Quit 51 prescription were as follows; uptake rate – 80.1%, 18.7% and 0.4% (see Table 2); cost per user - £115.45, £190.16 and £122.99 for NRT (see Table 3); relative success rate (against baseline of 4% cessation) – 2.14, 2.24 and 1.6, for varenicline and bupropion respectively in each case [4]. The figures are presented to cover various time horizons – 2 years (short term); 5–10 years (medium term) and lifetime horizon (long term). The Quit 51 approach produced an average of 35.21 quitters per 1000 smokers. This translated to health gains of 1.5 QALYs per 1000 clients in the short term to 23.4 QALYs per 1000 clients using a lifetime horizon. Although the cost per QALY gain in the first 2 years of Quit 51 (£58,736) is well above the NICE threshold of £20,000 per QALY, this intervention would become cost-effective in the medium term and would be highly cost-effective in the long term, compared with the counterfactual scenario. For every £1 invested in Quit 51, over a lifetime horizon one would expect a return of 65p just in healthcare savings (i.e. savings coming from treating fewer cases of smoking attributable diseases) but this would increase to a more substantial £4.22 if QALY gains are considered from a societal perspective.

**Table 4:** Short-, medium- and long-term costs and outcomes of the Quit 51 approach modelled using the EQUIPTMOD model [22] based on 35.21 quitters per 1000 smokers

Metrics	Time horizon			
	2 years	5 years	10 years	Lifetime
Average healthcare costs (per smoker)	£862	£2091	£3944	£11,608
Average total costs (per smoker)(includes cost of intervention)	£957	£2186	£4039	£11,704
Average QALYs (per smoker)	1.57	4.53	6.57	14.81
QALY gains (per 1000)^a^	1.5	3.7	7.5	23.4
Cost/QALY^a^	£58,736	£19,756	£7582	£402
Benefit-Cost ratio^a^(healthcare savings only)	0.07	0.16	0.29	0.65
Benefit-Cost ratio^a^ (healthcare savings + QALY gains valued at £20,000/QALY)	0.29	0.73	1.44	4.22

^a^Compared with a baseline scenario of no intervention

## Discussion

In this study we have used observational data recorded by a smoking cessation service to gain an understanding of the typical real world CPQs from a service or NHS perspective. From these data, we estimate the average cost per quitter at £403.59, with a significant degree of variation seen across certain subgroups of the client population. These calculations were based on applying an average rate with respect to adviser time and a more accurate picture may be obtained where more detailed individual-level information exists. Furthermore, Quit 51 overhead costs were not included in addition to there being no allowance made for costs to the individual of service use (travel and parking costs etc.). Thus the figures presented here should be interpreted strictly in relation to costs associated with provision of medication and behavioural support by stop smoking services.

Age-based results imply greater difficulty amongst the young (teenagers and the 20–29 year old group in this instance) in quitting, as has been reported elsewhere [28]. This age group is an important one to reach and support, given that the long term benefits of cessation are greater at this stage of life than amongst those of an older age [1]. Analysis elsewhere has shown CPQ figures for different types of internet and mobile phone smoking cessation assistance [29] are favourable in relation to those presented here. Given widespread use of internet technology amongst the young in general, this represents an appealing alternative as a strategy for driving down quit rates (and associated costs) with users of the service in this age group.

These data show that average cost per quit amongst males was slightly reduced relative to females, a finding which is consistent with men being more likely to quit in the context of smoking cessation services [28, 30], although the magnitude of the difference was not great when considered against the overall average costs observed (difference = £18.70 at 12 weeks). If the reason behind the different quit rates between men and women was related to anxiety about weight gain due to quitting smoking, the provision of a more comprehensive service combining both smoking cessation and weight loss may be worth considering [31].

Although varenicline users had a higher quit rate than those using NRT, this didn’t translate to a lower CPQ, which was slightly higher for varenicline than NRT. Models and analysis looking into ICERs and QALYs elsewhere suggest varenicline is more cost-effective than NRT [15, 16, 32], thus contrasting to a degree with the findings in this analysis. The estimated CPQ for bupropion was perhaps surprisingly low, although it should be remembered that the standard course of treatment for bupropion users only amounts to three prescriptions to cover 8 weeks in contrast to NRT and varenicline, which are prescribed to cover a period of 12 weeks (see Table 1). Even allowing for this, the CPQ for bupropion was strikingly low which contrasts with results in the literature. Nonetheless, the sample for this subgroup was very low and thus associated figures should be considered indicative only.

Greater CPQ with increased nicotine dependence is a logical result of greater difficulty quitting with increased dependence. No obvious trend was evident in relation to the deprivation groups although it was notable that the lowest CPQ was observed in relation to the most deprived group. It is possible to speculate that the higher prevalence of smoking in the most deprived population may mean that there is a higher proportion in this group who have low levels of addiction compared to other social groups, where those who can easily give up have already done so and one is left with a rump of smokers who find quitting very difficult.

In this preliminary work, we have only considered CPQ across subgroups of individual factors without considering how they might covary together (e.g. age and gender). Exploratory work indicated that some kind of interaction may be present between gender and age group, but this was not within the scope of the current preliminary presentation. Nonetheless, this could provide the basis of future work, particularly if more detailed individual costings become available.

In this analysis, the calculation of CPQ was primarily restricted to a health service perspective. However, the true cost of quitting smoking also involves the smoker’s time and travel to and from service venues, and other indirect costs such as leave from work or provision of childcare. Therefore, the true cost to individuals and society is likely to be greater than the figures we have presented here.

Our analysis was based on routinely collected service data. It was necessary to exclude a proportion of the data before the calculation was carried out. In the case of exclusion on the basis of (i) date and (ii) quitter recorded as pregnant this was a practical necessity. In addition, some records were omitted as either no or multiple treatment was recorded (4254 of the original 15,682 records fell into this category). This raises the issue of data quality when dealing with data of this nature and potential bias introduced due to excluded and missing records. Furthermore, we have assumed the five CCGs considered are approximately representative of England as a whole. It is difficult to quantify the resulting impact but the large sample size provides some reassurance regarding robustness in these results. Furthermore, the bootstrapped confidence intervals allowed us to capture quantifiable uncertainty with a non-standard distribution.

Costings were based on figures provided by Quit-51 as of the financial year 2015–2016 and the results should be interpreted on this basis. The impact of inflation over this period could have been taken into account, but should not have greatly altered the findings, particularly in regard to relative differences between the subgroups of interest, as the inflation rate was low over the period of this study.

Our cost per QALY findings are subject to the limitations of EQUIPTMOD which have been described in Coyle et al. [22]. We made use of the available model to predict the value of Quit 51 against a baseline scenario of no intervention to make meaningful predictions – this assumption may however be an important limitation in itself. Information on quit rates at 52 weeks was recorded sparingly in this dataset and therefore we used the quit rates adopted within EQUIPTMOD. This means that we have assumed relapse rates of approximately 70%, 81% and 88% for the NRT, varenicline and bupropion treatment arms respectively subsequent to the observed 12-week quit rates in Quit 51. Thus these figures are based on a number of assumptions, but nonetheless serve to demonstrate how parameters derived from our calculations can be used to estimate long-term population level cost-benefit outcomes.

## Conclusions

This work has highlighted differences in CPQ between different demographic subgroups, most notably increased expense in relation to teenage and 20–29 year old clients, female users and those reporting greater nicotine dependence at baseline. Such information could be factored into service provision, based on user population characteristics. The higher costs associated with treating teenagers are particularly important from a service planning perspective. The CPQ for varenicline was higher than that for NRT and bupropion, which is in contradiction to some other studies. The data presented in this study may contribute to future meta-analyses to resolve the relative CPQ in real life situations.

## Abbreviations

CCG: Clinical commissioning group
CI: Confidence interval
CPQ: Cost per quit
FTND: Fagerstrom test for nicotine dependence
IMD: Index of multiple deprivation
NCSCT: National centre for smoking cessation treatment
NICE: National institute for health care and excellence
NRT: Nicotine replacement therapy
QALY: Quality adjusted life year
RCT: randomised controlled trial
VAT: Value added tax
